# Efficacy and safety of Xinnaoning capsule in treating chronic stable angina (qi stagnation and blood stasis syndrome): Study protocol for a multicenter, randomized, double-blind, placebo-controlled trial

**DOI:** 10.1097/MD.0000000000016539

**Published:** 2019-08-02

**Authors:** Jun-Nan Zhao, Ying Zhang, Xu Lan, Yao Chen, Jing Li, Ping Zhang, Li-Qi Wu, Shu-Ting Jia, Yue Liu, Feng-Qin Xu

**Affiliations:** a Institute of Geriatric Medicine, Xiyuan Hospital of China Academy of Chinese Medical Sciences; b Beijing Duheng for Drug Evaluation and Research Co., Ltd; c Cardiovascular Diseases Center, Xiyuan Hospital of China Academy of Chinese Medical Sciences, Beijing, China.

**Keywords:** chronic stable angina, qi stagnation and blood stasis, traditional Chinese medicine, Xinnaoning capsule

## Abstract

**Background:**

Chronic stable angina (CSA) is a cardiovascular disease with high prevalence. At present, drug treatment is still the main measure of stable angina pectoris. Traditional Chinese medicine has a long history in the treatment of CSA. Qi stagnation and Blood stasis syndrome is a common syndrome of CSA. Xinnaoning (XNN) capsule is considered as an effective adjuvant treatment for CSA with the efficacy of promoting qi and blood circulation but lack of high-quality clinical evidence. The purpose of this study is to evaluate the efficacy and safety of XNN capsule compared with placebo by clinical trial.

**Methods:**

This multicenter, randomized, double-blind, placebo-controlled trial will be conducted with a total of 240 participants diagnosed with chronic stable angina (qi stagnation and blood stasis syndrome). The participants will be randomized (1:1) into groups receiving either XNN or placebo for 12 weeks. After a 2-week run-in period, they will receive either XNN or placebo (3 pills, 3 times daily) for 12 weeks on the basis of conventional therapy. The primary outcomes include changes in the integral scores of angina symptoms. The secondary outcome measures include changes in the total score of traditional Chinese medicine syndrome, severity grading of angina pectoris, the number of angina pectoris per week, nitroglycerin dosage, score of seattle angina scale, serum homocysteine, incidence of cardiovascular events. Safety outcomes will also be assessed. Adverse events will be monitored throughout the trial.

**Results:**

This study will investigate whether XNN capsule can alleviate clinical symptoms, and improve quality of life of patients with chronic stable angina (qi stagnation and blood stasis syndrome). The results of this study will provide clinical evidence for the application of XNN capsule in the treatment of chronic stable angina.

**Trial registration:**

ClinicalTrials.gov: NCT 03914131.

## Abstract 2

Supplemental Digital Content is available in the text

## 1 Introduction

Cardiovascular disease (CVD) causes huge health and economic burden worldwide.^[1]^ Chronic stable angina (CSA) is a major symptomatic presentation in about half of the patients with coronary heart disease (CHD).^[2]^ In China, stable angina is a common disability disease with a prevalence of about 3.6%.^[3]^ CSA^[4]^ is a clinical syndrome of acute and transient hypoxic-ischemic myocardium caused by increased myocardial load on the basis of severe coronary artery stenosis. It is usually a temporary chest discomfort characterized by temporary poststernal crushing pain or suffocation (angina pectoris), which can be induced by exercise, mood fluctuations, or other stresses and relieved by rest or nitroglycerin.^[5]^

The current treatment for CSA mainly includes medication and revascularization. The purpose of medication includes improvement of symptoms, ischemia and long-term prognosis. Traditional Chinese Medicine (TCM) has a long history in managing CSA. Chinese patent medicines (CPMs) are widely used in China as substitutes and adjuvant therapies for western medicines.^[6]^ As an adjuvant treatment of conventional anti-angina therapy, CPMs play an active role in reducing the incidence of primary endpoint events, angina attack^[7]^. TCM combined with anti-anginal medications have been proved effective and safe in the treatment of CSA.^[8]^ According to TCM theory, the fundamental cause of chronic stable angina (qi stagnation and blood stasis syndrome) is qi stagnation and blood stasis. Xinnaoning (XNN) capsule is composed of *Ginkgo biloba* leaves, *Euonymus microphylla*, *Salvia miltiorrhiza*, ginger seed, and Xiebai, with the functions of promoting circulation of qi and blood, relieving pain.

However, the methodological quality of most previous studies is usually assessed as low. Although some Cochrane reviews have shown the potential benefit of TCM in treating CHD,^[9]^ more evidence from high-quality trials are needed to support the clinical use of Chinese patent medicine. A well designed randomized controlled trial can be used to provide high-level evidence for the clinical application of XNN capsule. Therefore, the purpose of this study is to clarify the efficacy and safety of XNN capsule in the treatment of CSA through multicenter, randomized, double-blind, placebo-controlled, clinical trial.

## 2 Methods

This research will evaluate the efficacy and safety of XNN capsule in adults with chronic stable angina (qi stagnation and Blood stasis syndrome) to clarify the role of XNN capsule in the medical field.

This is a multicenter, randomized, double-blind, placebo-controlled clinical trial. The study is registered at ClinicalTrials.gov (NCT03914131) and complies with the principles of the Declaration of Helsinki and Good Clinical Practice guidelines. The protocol design is based on the following Standard Protocol Items: Recommendations for Interventional Trials (SPIRIT) checklist (see additional file 1). We will rigorously follow the Consolidated Standards of Reporting Trials (CONSORT) Extension for Chinese Herbal Medicine Formulas 2017 recommendations in reporting the results.^[10]^

**Figure 1:**
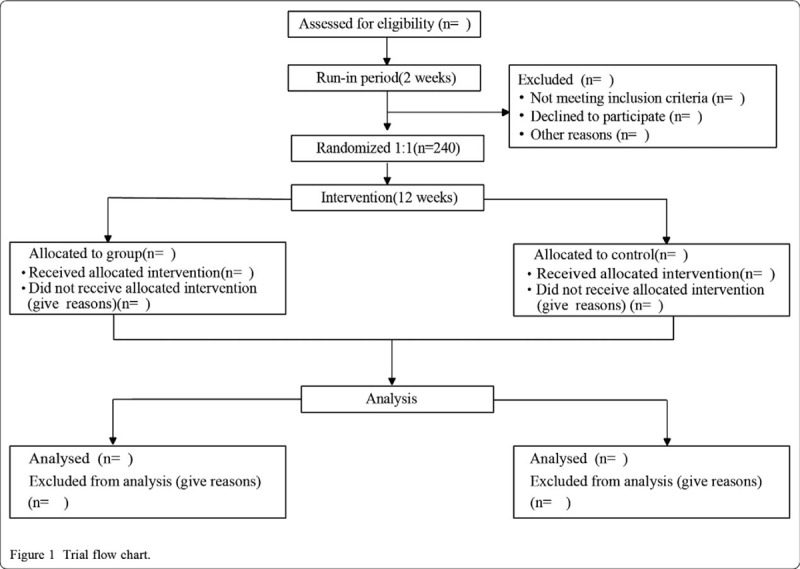
Trial flow chart.

The trial will be conducted at 9 centers in China (see additional file 2), the included subjects (n = 240) will be randomly divided into experimental (n = 120) and control groups (n = 120) according to a random number table, which is generated by SAS 9.1.3. Both groups will undergo a 2-week run-in period and a 12-week treatment period. A flow diagram of the trial is illustrated in Figure 1.

### 2.1 Research subjects

Research subjects are 240 patients who meet all of the following inclusion criteria and who have none of the listed exclusion criteria.

### 2.2 Diagnostic criteria

#### 2.2.1 Western medicine diagnostic criteria

The diagnostic criteria for CHD are:

(1) a history of myocardial infarction, with or without revascularization (percutaneous coronary intervention [PCI] or coronary artery bypass grafting) treatment;
(2) coronary angiography confirmation of stenosis greater than 50% of at least 1 major branch of the coronary artery luminal diameter, with or without revascularization treatment; the diagnostic criteria for chronic stable angina were determined according to the Chinese Medical Association's 2007 Guidelines for the Diagnosis and Treatment of Chronic Stable Angina,^[11]^ 2012 ACCF/AHA/ACP/AATS/PCNA/SCAI/STS Guideline for the Diagnosis and Management of Patients With Stable Ischemic Heart Disease,^[12]^ 2013 ESC guidelines on the management of stable coronary artery disease.^[13]^

The classification of angina referred to the Canadian Cardiovascular Society (CCS) Functional Classification of Angina ^[14]^ (see additional file 3).

#### 2.2.2 TCM diagnostic criteria

According to the technical guidelines for clinical research of TCM drugs and natural drugs treated for angina and coronary artery disease (2011).^[15]^ Primary symptoms are pain and stuffiness in the chest. Secondary symptoms are fullness in the chest and hypochondrium, palpitation, shortness of breath, cyanotic lips, fatigue and lassitude, dizziness, irascibility and quick in temper, bluish and purplish sublingual veins. Tongue characteristic is purplish tongue and pulse manifestation is choppy pulse. A patient with 1 of the primary symptoms and 2 of the secondary symptoms, combined with the tongue characteristic and pulse conditions, can be diagnosed as having qi stagnation and blood stasis syndrome.

### 2.3 Inclusion criteria

- A clear history of old myocardial infarction, or PCI, or coronary artery bypass graft (CABG);
- Coronary angiography (results suggesting at least 1 coronary stenosis with stenosis ≥50%) or coronary CTA suggesting luminal stenosis ≥50%;
- Those who met the diagnostic criteria of chronic stable angina pectoris: those who had a history of angina pectoris more than 1 month and had no significant changes in the degree, frequency, nature, and inducing factors of angina pectoris;
- The severity of angina pectoris of the CCS was classified as Grade I to Grade III, and angina pectoris occurred more than twice a week;
- TCM syndrome differentiation is qi stagnation and blood stasis syndrome;
- Age ranges from 30 to 79 years old;
- Before the beginning of the study, patient fully understands the study and is willing to sign the informed consent form.

### 2.4 Exclusion criteria

- Severe cardiopulmonary dysfunction (cardiac function III, IV, severe abnormal pulmonary function);
- Poor control of hypertension (systolic blood pressure >160mmHg or diastolic blood pressure >100mmHg after treatment);
- Complicated with liver and kidney function damage, ALT, AST (>1.5 times of the upper limit of normal value), or Cr (>the upper limit of normal value), combined with hematopoietic system and other serious primary diseases;
- Acute myocardial infarction within 3 months after interventional therapy;
- Cardiac pacemaker;
- Pregnancy, lactation or pregnancy planners;
- Anaphylactic constitution or allergic to known ingredients of research drugs;
- Chest pain caused by other causes (moderate anemia, hyperthyroidism, etc.)
- Those who participated in other clinical drug trials within 1 month;
- According to the judgment of the researchers, it is not advisable to participate in clinical researchers;
- Other factors affecting ST-T changes in electrocardiogram (ECG), such as myocardial hypertrophy, left bundle branch block, and so on.

### 2.5 Rejection criteria

#### 2.5.1 Termination by researchers

- The subject's condition becomes worse in the course of the study. If there were more episodes of angina pectoris, prolonged duration of each episode, or worsened pain, it is necessary to adjust the treatment, hospitalization or surgery and other emergency treatment measures;
- The subject has some complications or special pathophysiological changes, and it was not suitable to continue the study;
- The subject uses the drug prohibited by the program in the course of the study;
- If the subject's angina pectoris symptoms become worse in the course of the study, the investigator decided to take other treatments for angina pectoris or increase the dosage, and quit as an invalid case.

#### 2.5.2 Termination by subjects

According to the informed consent form, the subjects have the right to withdraw from the trial. The reasons will be specified and recorded. For any reason, the case record should be retained for the case with which the study was withdrawn, and the final test result will be carried forward to the final result.

- The subject is reluctant to take the trial drug or continue the trial;
- There is no medical record to be evaluated.

### 2.6 Suspension criteria

- If serious safety problems occur in the research, the research should be suspended in time;
- In the study, it was found that the clinical research program was difficult to evaluate the drug effect due to major mistakes; or it was difficult to evaluate the drug effect during the implementation of the program;
- The sponsor requests suspension (such as funding, management reasons, etc)
- The drug administration department cancels the research.

### 2.7 Treatment plan

#### 2.7.1 Routine treatment

Both the experimental group and control group can take antiplatelet drugs, angiotensin-converting enzyme inhibitor (ACEI) or angiotensin II receptor antagonist (ARB), statins, calcium ions antagonists, beta-blockers, long-acting nitrates, the above drugs can continue to be used without changing the original dose; nitroglycerin 0.5 mg can be taken sublingually when angina pectoris is intolerable; detailed records of the number of medications and the amount of medication;Exercise regulations: 3 to 5 times a week, each lasting 30 to 40 minutes, walking 6.5 to 7.5 kilometers per week.

#### 2.7.2 Introduction period

The participants will be allocated to experimental group or control group. The course of treatment was 14 weeks, in which the introduction period was 2 weeks (time window ± 2 days), and the introduction period medication regimen: both groups took XNN capsule simulator (Guizhou Jingcheng Pharmaceutical Co., Ltd.),3 capsules/time, 3 times/day, taken orally, after meals.

#### 2.7.3 The treatment period

Blind treatment period of 12 weeks (time window ± 4 days), treatment period medication program:

- Experimental group: Xin Nao Ning capsule (Guizhou Jingcheng Pharmaceutical Co., Ltd.),3 capsules/time, 3 times/day, taken orally, after meals;
- Control group: XNN capsule simulator (Guizhou Jingcheng Pharmaceutical Co., Ltd), 3 capsules/time, 3 times/day, taken orally, after meals.

### 2.8 Outcome

#### 2.8.1 Primary outcomes

The changes of curative effect of angina pectoris symptoms: The number of angina attacks, duration, degree of pain, and the dosage of nitroglycerin are used as indicators for scoring. Score ranges 0 to 15, the higher the score, the more severe the angina. (Table 1)

**Table 1:**
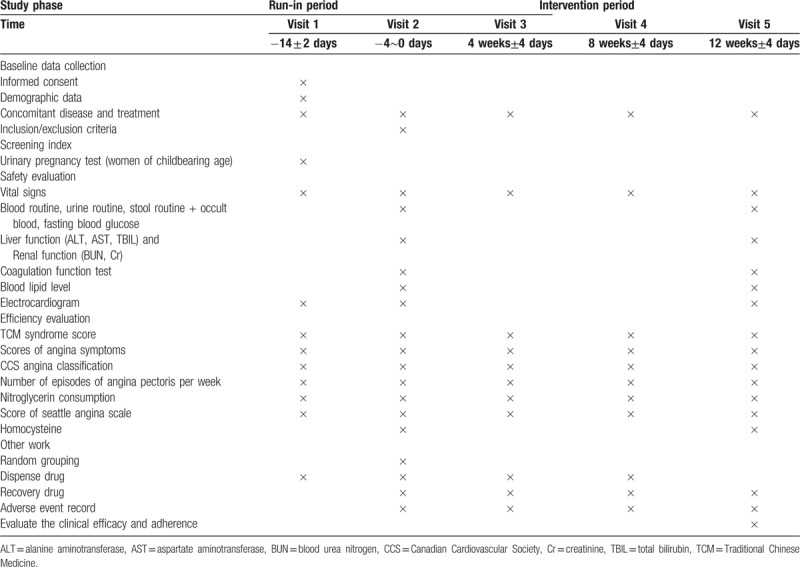
Measurement items and points of data capture.

#### 2.8.2 Secondary outcomes

- Therapeutic effect of TCM syndromes: The main symptoms of qi stagnation and blood stasis syndrome are chest pain, chest tightness, secondary symptoms such as palpitation, shortness of breath, chest swelling, fatigue, dark lips, dizziness, veins at the base of tongue, irritability, and so on. The severity and presence of these symptoms are used as criteria for scoring. Score ranges 0 to 28. The higher the score, the more serious it is.
- Grading changes of severity of angina pectoris: Reference to CCS Angina Severity Classification Standard. I: General physical activity does not cause angina pectoris, such as walking and going upstairs, but tension, rapid or sustained exertion can cause the onset of angina pectoris. II: Daily physical activity is slightly restricted. Walking fast or upstairs, climbing high, walking after meals or upstairs, walking in cold or wind, and emotional excitement can cause angina or only occur within a few hours after waking up. It is limited to walk more than 200 meters or climb stairs above 1 floor at normal speed. III: Daily physical activity is obviously limited, and angina pectoris can occur when walking 100 to 200m at normal speed or climbing a staircase. IV: Angina symptoms can occur when you are slightly active or at rest.
- Changes in the number of angina attacks per week: Frequency of angina pectoris episodes per week.
- Nitroglycerin dosage;
- Seattle Angina Questionnaire (SAQ): the Seattle Angina Questionnaire (SAQ) measured a total of L9 problems, including physical activity limitation, stable state of angina, frequency of angina attack, satisfaction with treatment, and knowledge of disease. A kind of Assessment of Seattle Scale: The Seattle Angina Scale was divided into 5 items and 19 items: Physical Activity Restriction (PL, Question 1), Angina Stable State (AS, Question 2), Angina Attack (AF, Question 3–4), Treatment Satisfaction (TS, Question 5–8), Disease Cognition (DS, Question 9–11), 19 items of 5 items and the total score of SAQ. The formula is transformed into standard integral, standard integral = (actual score−the lowest score in this respect)/(the highest score in this respect−the lowest score in this respect) ∗ 100, the higher the score, the better the quality of life and the state of body function of patients;
- Blood homocysteine: the changes of blood HCY before and after treatment are compared between the 2 groups;
- Incidence of cardiovascular events: Sudden cardiac death, acute myocardial infarction, heart failure, percutaneous transluminal coronary angioplasty, coronary artery bypass grafting, malignant arrhythmia, cardiogenic cerebrovascular accident, angina pectoris requiring hospitalization, and so on.

#### 2.8.3 Safety outcomes

- Possible symptoms of adverse reactions;
- Vital signs such as body temperature, pulse, respiration, blood pressure, and so on;
- Laboratory examination: blood routine,urine routine,stool routine + occult blood, liver function (ALT, AST, TBIL),renal function (BUN,Cr), Coagulation function test, fasting blood glucose, blood lipid;
- ECG.

#### 2.8.4 Evaluate points

The evaluation time point is shown in Table 1.

### 2.9 Sample calculation

The sample size was estimated to treat the symptoms of angina pectoris for 12 weeks as the main effect index according to the statistical requirements. According to the literature, the total effective rate of basic treatment + placebo was 67.5%, and the total effective rate of basic treatment + test drug was estimated to be 84.3. %, let α = 0.05, 1-β = 0.2, according to the test group: control group = 1:1 set, after calculation, the minimum sample size that meets the statistical requirements is 98.4 cases per group, considering no more than 20% the shedding rate was 120 cases in each group, a total of 240 cases.

### 2.10 Implementation management and quality control

#### 2.10.1 Randomization

Randomized sequences will be generated by the contract research organization (Beijing Duheng for Drug Evaluation and Research Co., Ltd.) as the third party. A random arrangement (i.e., a random coding list) of 240 subjects received (experimental and control) was generated by SAS 9.1.3 statistical software. According to the randomly assigned drug number assigned by each hospital, each center is assigned a connection coded drug that is connected to each other. The administrator will dispense drugs 1 by 1 according to the order of each subject's visiting and drug number from small to large.

#### 2.10.2 Allocation concealment

The random number is sealed with an opaque envelope and is managed centrally by each test center.

#### 2.10.3 Blingding

All patients and researchers will be blinded to the treatment assignments until the study is completed. Duplicated blinding codes will be provided to the Clinical Research Institution of Drugs in Xiyuan Hospital, Chinese Academy of Traditional Chinese Medicine and the blinding codes cannot be broken until all the clinical data are entered into a database and locked, except in an emergency situation.

#### 2.10.4 Emergency unblinding

In case of an emergency (such as a serious adverse event) or when a patient needs to be rescued and must know what treatment the patient receives, the principal investigator should be notified immediately and the emergency letter should be opened urgently. Once the emergency letter is opened, the numbered subjects will withdraw from the study. Researchers should record the causes in the case report form, and record the causes, time and place of emergency blindness detection on the emergency letter, with at least 2 signatures. After the study, it will be retrieved with the case report form.

#### 2.10.5 Adverse events

All adverse events (AEs) must be evaluated objectively. When an adverse event occurs, the investigator decides whether to stop the study or not based on the diagnosis and treatment of adverse events. The unit undertaking the clinical trial immediately takes necessary measures to protect the safety of the subject. If the trial lasted for more than 4 weeks, the subject was withdrawn due to increased severity (increased number of episodes, prolonged duration of episodes, increased pain, increased nitroglycerin use),such cases are included as statistically ineffective cases.

#### 2.10.6 Serious AEs

If serious adverse events occur in the study, including hospitalization, prolonged hospitalization, disability, affect on the ability to work, life endangerment, or death, the unit undertaking the clinical trial shall immediately take the necessary measures to protect the safety of the subject and report to the local provincial drug regulatory authority and the State Food and Drug Administration, the sponsor, within 24 hours.

### 2.11 Data management

The data will be recorded into case report forms (CRFs) in time and accurately. The 2 data administrators independently perform double entry and proofreading. For the questions in the CRFs, the data administrator will fill in the Question Answer Form (DRO) and send an inquiry to the researcher. Answer and return, the data administrator will modify, confirm and enter the data according to the researcher's answer, and if necessary, the DRO can be issued again. After blindly reviewing and confirming that the database is built correctly, the database is locked by the main investigator, sponsor, and statistical analyst.

### 2.12 Statistical analysis

SPSS 22.0 statistical software will be used for statistical analysis. The measurement data were described by means of mean ± standard deviation, and the paired *t* tests was used to compare the intra-group differences before and after treatment. The count data were statistically described using the frequency and the changes before and after treatment were analyzed by Chi-square tests or non-parametric tests. Analysis of variance (ANOVA) or Chi-square tests were used to compare demographic data and other baseline indicators to measure the balance between the 2 groups. The primary and secondary indicators were analyzed by full analysis set (FAS) and per protocol set (PPS). The effectiveness analysis used the CMH test to consider the central effect to analyze the efficacy of the 2 groups. If there are related factors that significantly affect the efficacy, these factors should be considered as covariates when comparing the efficacy of the 2 groups. Safety analysis will be used Chi-square tests to compare the incidence of adverse events.

## 3 Discussion

This study is a randomized, double-blind, placebo-controlled trial designed for XNN capsule to evaluate the efficacy and safety in the treatment of chronic stable angina. In this study, we use software (SAS9.1.3) to generate random sequences and hide random numbers in opaque envelopes, which may avoid potential selective bias. The blind method of participants and researchers aims at eliminating the subjective bias and personal preferences. Due to the key characteristics of Chinese herbal medicine such as color, odor, taste, and others, placebo control is limited in past clinical studies. However, with the progress of pharmaceutical technology, we can strictly implement placebo control in this study. For clinical trials, placebo-controlled trials can reliably demonstrate the efficacy of research drugs and can distinguish whether adverse events are caused by drugs or underlying diseases. At the same time, placebo-controlled trials are more efficient and require a smaller number of subjects. Moreover, it is expected to increase the ability of detecting the true efficacy of drugs. If the placebo effect is not strictly controlled, the effect of TCM will be exaggerated. It is worth mentioning that all subjects in this study received basic treatment routinely, and the use of placebo did not affect the normal treatment of subjects, which met the ethical requirements. This method has been widely used in clinical research of TCM.

Second, we focus our research on the improvement of patients’ symptoms, it is more and more important in the evaluation of the efficacy on CSA.^[16]^ Guidelines for the treatment of chronic stable angina also suggest that treatment objectives should include improvement of symptoms.^[12]^ TCM is considered as a potential choice to alleviate symptoms and improve the quality of life. Therefore, we regard the efficacy of angina pectoris symptoms and TCM syndromes as therapeutic indicators, because they are related to patients’ symptoms and quality of life.

As a certified Chinese patent medicine that is used to treat for chronic stable angina (qi stagnation and blood stasis syndromes), XNN capsule (Z20025697) is mainly composed of Ginkgo biloba leaves (Yinxingye), Salvia miltiorrhiza (Danshen), Ginger seeds of Big Fruit (Daguomujiangzi), Euonymus microphylla (Xiaoyehuangyang) and Allium macrophylla (Xiebai),with the efficacy of promoting qi and blood circulation. In addition, previous studies^[17–20]^ have shown that XNN capsule is safe and effective in cardiovascular protection.XNN capsule is considered as an effective adjuvant treatment for chronic stable angina, so we designed this trial to confirm that XNN capsule with conventional treatment can alleviate symptom in patients with chronic stable angina (qi stagnation and blood stasis syndrome).

However, this study also has some limitations, 1 of which is the lack of evaluation on the long-term prognosis of XNN capsule. In this study, the treatment cycle was 12 weeks, which was relatively short. Due to the short follow-up time and limited budget, the potential role of XNN capsule in reducing acute cardiovascular events and all-cause mortality remains unclear. In addition, XNN capsule is the representative drug of “ Therapy of Heart and Brain Co-treatment”, but the incidence of cerebrovascular events and cognitive function are not involved in this study. Therefore, a randomized controlled trial should be designed in the future to compare XNN capsule with traditional therapy, prolong the follow-up period and add the evaluation of efficacy in cerebrovascular and cognitive function.

## 4 Others

### 4.1 Ethic issue

This trial has been reviewed and approved by the Ethics Committee of Xiyuan Hospital affiliated with the China Academy of Chinese Medical Sciences (approval number: 2017XL027-2). Researchers are responsible for ensuring that the study is conducted in accordance with the principles of the Declaration of Helsinki and GCP. Participants will voluntarily provide their written informed consent before any study procedures, and they can voluntarily withdraw from the study for any reason.

### 4.2 Trial status

Currently, participant recruitment is ongoing.

### 4.3 Consent for publication

We will give informed consent for the publication of the dataset from patients at the point of recruitment to the trial. All the patient details will be fully anonymous.

## Acknowledgments

The authors thank those who have participated in the trial. They are also grateful to the staff of the participating centers.

## Author contributions

**Conceptualization:** Fengqin Xu.

**Data curation:** Jun-Nan Zhao, Xu Lan, Yao Chen.

**Investigation:** Ying Zhang, Jing Li, Ping Zhang, Li-Qi Wu, Fengqin Xu.

**Methodology:** Jun-Nan Zhao, Yue Liu.

**Project administration:** Feng-Qin Xu.

**Supervision:** Shu-Ting Jia.

**Writing – original draft:** Jun-Nan Zhao, Ying Zhang.

**Writing – review & editing:** Yue Liu, Feng-Qin Xu.

## Supplementary Material

Supplemental Digital Content
